# Regions of ryanodine receptors that influence activation by the dihydropyridine receptor β_1a_ subunit

**DOI:** 10.1186/s13395-015-0049-3

**Published:** 2015-07-22

**Authors:** Robyn T. Rebbeck, Hermia Willemse, Linda Groom, Marco G. Casarotto, Philip G. Board, Nicole A. Beard, Robert T. Dirksen, Angela F. Dulhunty

**Affiliations:** Department of Biochemistry, Molecular Biology and Biophysics, University of Minnesota, Minneapolis, MN USA; John Curtin School of Medical Research, Australian National University, Canberra, Australian Capital, PO Box 334, Canberra, ACT 2601 Australia; Department of Physiology and Pharmacology, University of Rochester Medical Center, Rochester, NY USA; Discipline of Biomedical Sciences, Centre for Research in Therapeutic Solutions, University of Canberra, Canberra, ACT 2601 Australia

**Keywords:** Excitation-contraction coupling, Dihydropyridine receptor β_1a_ subunit, Ryanodine receptor isoforms, Skeletal muscle, Cardiac muscle

## Abstract

**Background:**

Although excitation-contraction (EC) coupling in skeletal muscle relies on physical activation of the skeletal ryanodine receptor (RyR1) Ca^2+^ release channel by dihydropyridine receptors (DHPRs), the activation pathway between the DHPR and RyR1 remains unknown. However, the pathway includes the DHPR β_1a_ subunit which is integral to EC coupling and activates RyR1. In this manuscript, we explore the isoform specificity of β_1a_ activation of RyRs and the β_1a_ binding site on RyR1.

**Methods:**

We used lipid bilayers to measure single channel currents and whole cell patch clamp to measure L-type Ca^2+^ currents and Ca^2+^ transients in myotubes.

**Results:**

We demonstrate that both skeletal RyR1 and cardiac RyR2 channels in phospholipid bilayers are activated by 10–100 nM of the β_1a_ subunit. Activation of RyR2 by 10 nM β_1a_ was less than that of RyR1, suggesting a reduced affinity of RyR2 for β_1a_. A reduction in activation was also observed when 10 nM β_1a_ was added to the alternatively spliced (ASI(−)) isoform of RyR1, which lacks ASI residues (A3481-Q3485). It is notable that the equivalent region of RyR2 also lacks four of five ASI residues, suggesting that the absence of these residues may contribute to the reduced 10 nM β_1a_ activation observed for both RyR2 and ASI(−)RyR1 compared to ASI(+)RyR1. We also investigated the influence of a polybasic motif (PBM) of RyR1 (K3495KKRRDGR3502) that is located immediately downstream from the ASI residues and has been implicated in EC coupling. We confirmed that neutralizing the basic residues in the PBM (RyR1 K-Q) results in an ~50 % reduction in Ca^2+^ transient amplitude following expression in RyR1-null (*dyspedic*) myotubes and that the PBM is also required for β_1a_ subunit activation of RyR1 channels in lipid bilayers. These results suggest that the removal of β_1a_ subunit interaction with the PBM in RyR1 could contribute directly to ~50 % of the Ca^2+^ release generated during skeletal EC coupling.

**Conclusions:**

We conclude that the β_1a_ subunit likely binds to a region that is largely conserved in RyR1 and RyR2 and that this region is influenced by the presence of the ASI residues and the PBM in RyR1.

**Electronic supplementary material:**

The online version of this article (doi:10.1186/s13395-015-0049-3) contains supplementary material, which is available to authorized users.

## Background

Contraction in skeletal and cardiac muscle depends on Ca^2+^ release from the intracellular sarcoplasmic reticulum (SR) Ca^2+^ store through ryanodine receptor (RyR) Ca^2+^ release channels embedded in the SR membrane. This Ca^2+^ release is crucial to excitation-contraction (EC) coupling. During EC coupling, cardiac RyRs (RyR2) are activated by an influx of extracellular Ca^2+^ through depolarization-activated dihydropyridine receptor (DHPR) L-type channels located in the surface and transverse-tubule membranes. In contrast, EC coupling in skeletal muscle is independent of extracellular Ca^2+^, apparently requiring a physical interaction between skeletal isoforms of the RyR (RyR1) and DHPR [1, 2]. However, despite exhaustive investigation, the physical components of this interaction still remain unclear [3, 4] and are investigated in this manuscript.

It is well established that the skeletal isoforms of both the membrane spanning α_1S_ subunit and the cytoplasmic β_1a_ subunit of the DHPR heteropentamer are essential for skeletal EC coupling [5, 6]. The α_1S_ subunit contains the voltage sensor for EC coupling [7, 8] and the “critical” region for skeletal EC coupling (residues L720-764/5) in its intracellular II-III loop [9–11]. The β_1a_ subunit is responsible for the targeting of the DHPR to the triad and assembly into tetrads that are closely aligned with RyR1 in the SR [12–14]. There is also evidence that the β_1a_ subunit also plays an active role in the EC coupling process. The β_1a_ subunit directly activates RyR1 channels incorporated into lipid bilayers and enhances voltage-activated Ca^2+^ release in skeletal muscle fibers [5, 15, 16]. The C-terminal region of β_1a_ (V490-M524) supports β_1a_ binding to RyR1 in vitro and influences voltage-induced Ca^2+^ release in mouse myotubes [15, 17, 18]. A peptide corresponding to the same residues mimics full length β_1a_ subunit activation of RyR1 channels in lipid bilayers [15] and a truncated peptide of the same region enhances voltage-induced Ca^2+^ release to the same degree as the full length β_1a_ subunit in intact adult mouse muscle fibers [16, 19]. Furthermore, overexpression of a β subunit interacting protein, Rem, in adult mouse skeletal muscle fibers was recently shown to reduce voltage-induced Ca^2+^ transients by ~65 % without substantially altering α_1S_ subunit membrane targeting or intramembrane gating charge movement or SR Ca^2+^ store content [20]. This suggests that the DHPR-RyR1 interaction may be uncoupled by virtue of direct interference of β_1a_ subunit. Residues in RyR1 that influence binding to the β_1a_ subunit have also been identified. The M3201-W3661 fragment of RyR1 binds to β_1a_ and the strength of binding is substantially reduced by replacing the six basic residues in a polybasic motif (PBM; K3495KKRRDGR3502) with glutamines [18]. Replacement of the same six residues with glutamines in the full-length RyR protein substantially reduces depolarization-dependent Ca^2+^ release [18]. The in vitro studies indicate a high-affinity interaction between the isolated RyR1 and the β_1a_ subunit that is influenced by the PBM. However, the basic residues unlikely bind directly to the hydrophobic residues in the β_1a_ C-terminus, although they could contribute to the overall conformation of the binding domain [21]. Similarly, it is unlikely that basic residue binding to the hydrophobic residues could contribute to EC coupling, although both basic residues and hydrophobic residues in the β_1a_ C-terminus influence EC coupling [16, 19].

The fact that skeletal DHPR and RyR isoforms are critical for skeletal-type EC coupling [22–26] suggests that isoform-specific regions of these proteins enable unique interactions in skeletal muscle. Also, in the context of isoform dependence, we reported that an alternatively spliced region of RyR1 (A3481-Q3485), located close to the PBM, is significant in setting the gain of EC coupling [27]. It is notable that RyR2 lacks the equivalent sequence to the ASI residues in ASI(+)RyR1 and, in this respect, more closely resembles the ASI(−)RyR1 isoform. Therefore, here we examined the RyR isoform dependence of the in vitro interaction with the β_1a_ subunit. We use the RyR isoforms as tools to explore regions of the RyR1 that influence its interaction with the C-tail of the β_1a_ subunit. Interactions between RyR2 and the cardiac β subunit were not examined as they have no physiological significance, and there is little sequence homology between the C-terminal tails of the cardiac and skeletal β isoforms [12–14].

The results indicate that while β_1a_ activates RyR1 and RyR2 isolated from the skeletal muscle and the heart and activates recombinant ASI(−)RyR1 and ASI(+)RyR1, β_1a_ activation of RyR2 and AS1(−)RyR1 requires higher β_1a_ concentrations than that required to activate RyR1 or ASI(+)RyR1. In addition, we show that neutralization of the basic residues in the RyR1 PBM abolishes β_1a_ activation of RyR1 in lipid bilayers and confirm that this also markedly reduces voltage-dependent Ca^2+^ release in skeletal myotubes. Together, the results reinforce the conclusion that β_1a_ binding to RyR1 contributes to EC coupling and suggest that the region encompassing the adjacent ASI residues and PBM is a determinant of β_1a_ binding to and regulation of RyR1.

## Methods

The work was approved by The Australian National University Animal Experimentation Ethics Committee (Australian Capital Territory, Australia) and by the University Committee on Animal Resources at the University of Rochester (New York, USA).

### Preparation of RyR1 ASI (−) and K-Q cDNA

The ASI(−)RyR1 variant was introduced into rabbit RyR1 cDNA (accession #X15750) using two-step site-directed mutagenesis as described previously [28]. The K-Q mutant (K3495KKRRGDR3502) was similarly introduced into a rabbit RyR1 cDNA by two-step site-directed mutagenesis in the following manner: using a BsiWI/BamHI subclone of RyR1, residues R3498Q and R3499Q were introduced via mutagenesis to create a double mutation (R3498Q/R3499Q). This mutant was used as a template to introduce a third mutation R3502Q. Finally, glutamine substitutions for residues K3495, K3496, and K3497 were introduced into the triple mutated plasmid to generate the PBM mutant Q3495QQQQGDQ3502 (K-Q mutant). The entire PCR-modified cDNA portion of the BswiWI/BamHI mutant subclone was confirmed by sequence analysis and then cloned back into full-length RyR1.

### Preparation of SR vesicles

Skeletal muscle SR vesicles were prepared from back and leg muscles (fast twitch skeletal muscle) from New Zealand white rabbits [29–31] and cardiac SR vesicles collected from sheep hearts [32, 33]. Vesicles were stored at −70 °C.

### Transfection and preparation of microsomal protein

Microsomal vesicles were collected from HEK293 transfected with recombinant rabbit RyR1 ASI(+), ASI(−), or K-Q RyR1 mutant cDNAs in mammalian expression vector (pCIneo) as described previously [28] with minor modifications. HEK cells were grown in 175-mm^2^ flasks at 37 °C, 5 % CO_2_ in 10 % fetal calf serum in MEM. At 50–60 % confluence, cells were transfected with 80 μg cDNA in a phosphate buffer solution (125 mM CaCl_2_, 70 mM NaH_2_PO_4_, 140 mM NaCl, 76 mM HEPES, 7 mM Na_2_HPO_4_, pH 7.2) using a calcium phosphate precipitation method. Cells were maintained for 48 h and then harvested in phosphate buffer (137 mM NaCl; 7 mM Na_2_HPO_4_; 2.5 mM NaH_2_PO_4_.H_2_O; and, 2 mM EGTA, pH 7.4). The pellet was resuspended in *homogenizing buffer* (300 mM sucrose, 5 mM imidazole, 1× complete EDTA-free protease inhibitor cocktail, pH 7.4), homogenized and centrifuged at 11,600 × *g* for 20 min. The resulting pellet was resuspended in homogenizing buffer, further homogenized and centrifuged at 91,943 × *g* for 2 h. The pellet was resuspended in homogenizing buffer, homogenized, and then briefly sonicated. The microsomal mixture was separated into 15 μL aliquots and stored at −70 °C.

### Preparation and injection of dyspedic myotubes

Primary cultures of myotubes were obtained from skeletal myoblasts isolated from newborn RyR1-null (*dyspedic*) mice as previously described [34, 35]. Four to 6 days after initial plating of myoblasts, nuclei of *dyspedic* myotubes were microinjected with cDNAs encoding CD8 (0.1 μg/μl) and the appropriate RyR1 expression plasmid (0.5 μg/μl) [36]. Expressing myotubes were identified 2–4 days after cDNA microinjection by incubation with CD8 antibody beads (Dynabeads, Dynal USA). All animals were housed in a pathogen-free area at the University of Rochester and experiments performed in accordance with procedures reviewed and approved by the local University Committees on Animal Resources.

### Preparation of β_1a_ subunit

The β_1a_ protein was expressed in transformed *Escherichia coli* BL21(DE3) and purified as described previously [15]. The proteins were dialyzed against a phosphate buffer (50 mM Na_3_PO_4_, 300 mM NaCl, pH 8) and stored at −70 °C.

### Single-channel recording and analysis

Channels from cardiac, skeletal, or HEK293 microsomal vesicles were incorporated into lipid bilayers with solutions containing (mM): *cis* (20 CsCl, 230 CsCH_3_O_3_S, 10 TES, and 1 CaCl_2_) and *trans* (20 CsCl, 30 CsCH_3_O_3_S, 10 mM TES, and 1 CaCl_2_), pH 7.2. After RyR incorporation, 200 mM CsMS was added to the *trans* solution for symmetrical [Cs^+^]. BAPTA was added to the *cis* solution as determined with a Ca^2+^ electrode to achieve 10 μM Ca^2+^, and 2 mM ATP was added. Bilayer potential, V_cis_-V_trans_, was switched between −40 and +40 mV. Channel activity under each condition was analyzed over 180 s using the program Channel 2 (developed by P. W. Gage and M. Smith). Threshold levels for channel opening were set to exclude baseline noise at ~20 % of the maximum single-channel conductance and open probability (*P*_*o*_), mean open time (*T*_*o*_), and closed open time (*T*_*c*_) measured. Dwell-time distributions for each channel were obtained using the log-bin method [37–39]. Event frequency (probability) was plotted against equally spaced bins (on a logarithmic scale) for open or closed durations (seven bins per decade). The time constants are indicated by the frequency peaks. The area under each peak indicates the fraction of single-channel open or closed events falling into each time constant component.

### Simultaneous measurements of macroscopic Ca^2+^ currents and transients in myotubes

The whole-cell patch clamp technique was used to simultaneously measure voltage-gated L-type Ca^2+^ currents (L currents) and Ca^2+^ transients in expressing myotubes [36]. Patch clamp experiments were conducted using an external solution consisting of (in millimolar): 145 TEA-Cl, 10 CaCl_2_, and 10 HEPES, pH 7.4 with TEA-OH and an internal pipette solution consisting of (in millimolar): 145 Cs-aspartate, 10 CsCl, 0.1 Cs_2_-EGTA, 1.2 MgCl_2_, 5 Mg-ATP, 0.2 K_5_-fluo-4, and 10 HEPES, pH 7.4 with CsOH. Peak L-current magnitude was normalized to cell capacitance (pA/pF), plotted as a function of the membrane potential (*I*-*V* curves in Fig. 6c), and fitted according to:

$$ I = {G}_{\mathbf{max}}*\ \left({V}_m - {V}_{\mathbf{rev}}\right)\ /\ \left(1 + \exp \left[\left({V}_{\mathbf{G1}/\mathbf{2}} - {V}_m\right)\ /\ {k}_G\right]\right) $$

where *G*_max_ is the maximal L-channel conductance, *V*_*m*_ is test potential, *V*_rev_ is the L-channel reversal potential, *V*_G1/2_ is the potential for half-maximal activation of *G*_max_, and *k*_*G*_ is a slope factor. Relative changes in fluo-4 fluorescence (ΔF/F) were measured at the end of each 200-ms depolarization, plotted as a function of the membrane potential, and fitted according to:

$$ \varDelta F/F = \left(\varDelta F\ /\ {F}_{\mathbf{max}}\right)/\left\{1 + \exp\ \left[\left({V}_{\mathbf{F1}/\mathbf{2}}-{V}_m\right)\ /\ {k}_F\right]\right\} $$

where Δ*F/F*_max_ is the maximal fluorescence change, *V*_F1/2_ is the potential for half-maximal activation of Δ*F/F*_max_, and *k*_*F*_ is a slope factor. The bell-shaped voltage dependence of ΔF/F measurements obtained in RyR1 K-Q mutant-expressing myotubes were fitted according to the following equation:

$$ \varDelta F/F = \left({\left(\varDelta F\ /\ F\right)}_{\mathbf{max}}\left(\left({V}_m - {V}_{\mathbf{rev}}\right)/k^{\prime}\right)\right)/\left(1 + \exp\ \left(\left({V}_{\mathbf{F1}/\mathbf{2}}-{V}_m\right)\ /\ {k}_F\right)\right) $$

where (Δ*F/F*)_max_, *V*_*m*_, *V*_rev_, *V*_F1/2_, and *k*_*F*_ have their usual meanings. The additional variable *k*′ is a scaling factor that varies with (Δ*F/F*)_max_ [40, 41]. The maximal rate of voltage-gated SR Ca^2+^ release was approximated from the peak of the first derivative of the fluo-4 fluorescence trace (dF/dt) elicited during the test depolarization at 30 mV. Pooled current-voltage (*I*-*V*) and fluorescence-voltage (Δ*F/F*-*V*) data in Table 1 are expressed as mean ± SEM.

**Table 1:** Parameters of fitted *I*-*V* and [ΔF/F]-V curves

	*I*-*V* data				[∆*F/F*]-*V* data		
	*G*_max_ (nS/nF)	*k* (mV)	*V*_half_ (mV)	V_rev_ (mV)	(∆*F/F*)_max_	*k* (mV)	*V*_half_ (mV)
WT RyR1 (*n* = 12)	264 ± 16	5.4 ± 0.4	10.5 ± 1.8	71 ± 1.8	3.4 ± 0.7	3.9 ± 0.5	-4.7 ± 1.6
RyR1 K-Q (*n* = 10)	201 ± 17*	5.7 ± 0.3	11.5 ± 1.7	70 ± 2.1	1.6 ± 0.3*	4.1 ± 04.	-2.5 ± 1.7

Maximal L-channel conductance (*G*_max_), the potential for half-maximal *G*_max_ (*V*_half_), slope factor (*k*), and reversal potential (*V*_rev_). Values presented as mean ± SEM for *I-V* data presented in Fig. 6c. Maximal Ca^2+^ transient [(*ΔF*/*F*)_max_], the potential at half maximal fluorescence (*V*_half_) and slope factor (*k*). Values presented as mean ± SE for [Δ*F/F*]-*V* data presented in Fig. 6d

*p < 0.05 vs WT RyR1-expressing dyspedic myotubes

### Immunofluorescence labeling

RyR-null (*dyspedic*) myotubes expressing either WT RyR or RyR K-Q mutant that were plated on glass coverslips were fixed and immunostained with a mouse monoclonal anti-RyR antibody (34C, 1:10; Developmental Studies Hybridoma Bank) and a sheep polyclonal anti-DHPR antibody (1:200; Upstate Biotechnology) overnight at 4 °C as previously described [41]. On the following day, coverslips were washed with PBS three times each for 5 min and then incubated for 1 h at room temperature in blocking buffer containing a 1:500 dilution of Alexa Fluor 488–labeled donkey anti-mouse IgG (Molecular Probes) and 1:500 dilution of rhodamine-labeled donkey anti-sheep IgG (Jackson ImmunoResearch Laboratories Inc.) and washed with PBS (three times for 5 min each). Coverslips were mounted on glass slides and images obtained using a Nikon Eclipse-C1 confocal microscope (Nikon Instruments Inc.) and a 40× oil objective. All confocal images were sampled at a spatial resolution (pixel diameter) of 100 nm.

### Statistics

Average data are given as the mean ± SEM. Statistical significance was evaluated by a paired or unpaired two-way Student’s *t*-test or analysis of variance (ANOVA) with Fisher’s post hoc test, as appropriate. The numbers of observations (*N*) are given in the figure legends. To reduce the effects of variability in control single-channel activity parameters (*P*_*oC*_, *T*_*cC*_, *T*_*oC*_) and to evaluate parameters after β_1a_ subunit (*P*_*oB*_, *T*_*cB*_, *T*_*oB*_) addition, data were expressed as the difference between the logarithmic values, i.e., log_10_ rel *P*_*o*_ = log_10_*P*_*oB*_–log_10_*P*_*oC*_. The difference from control was assessed with a paired *t*-test applied to log_10_*P*_*oC*_ and log_10_*P*_*oB*_. Variance in *P*_*o*_ parameter values was assessed with an unpaired *t*-test. A *p* value of <0.05 was considered significant.

## Results

### Ability of the β_1a_ subunit to activate different RyR isoforms

#### The β_1a_ subunit activates RyR1 and RyR2 channels

**Fig. 1:**
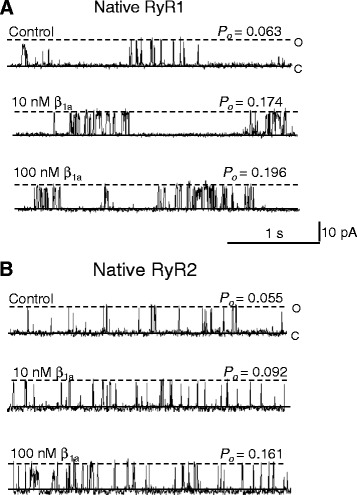
β_1a_ subunit increases RyR1 and RyR2 channel activity in lipid bilayers. **a** and **b** Three second (3 s) traces of representative activity from native (**a**) RyR1 or (**b**) RyR2 channels recorded at a test potential of +40 mV. Openings are shown as upward inflections from the closed (*c*) state to the maximum open (*o*) level. Results are shown before (top panel; control, *cis* 10 μM [Ca^2+^] and 2 mM ATP) and after addition of 10 nM β_1a_ subunit (*middle* panel) and then 100 nM β_1a_ subunit (*bottom* panel) to the *cis* chamber. Open probability (*P*_*o*_) is shown at the right hand corner of each trace

**Fig. 2:**
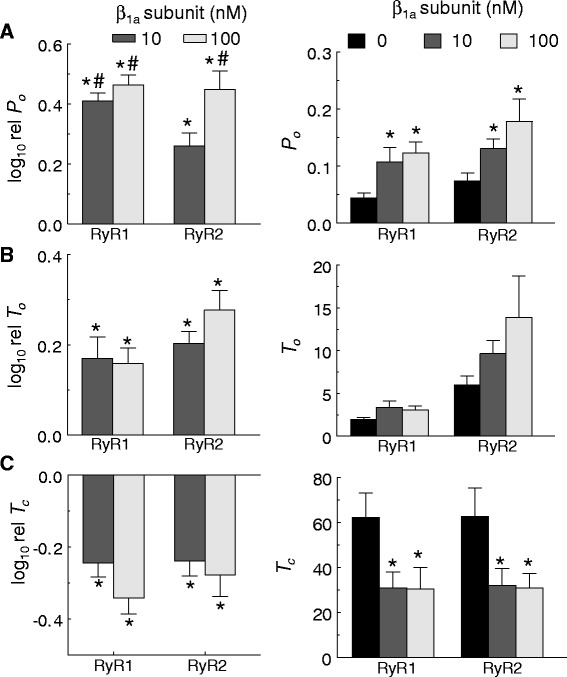
β_1a_ subunit increases RyR1 and RyR2 channel activity in lipid bilayers. Single-channel gating parameters of RyR1 and RyR2 in response to 10 or 100 nM β_1a_ subunit. **a** (*left*) Average relative *P*_*o*_ (log_10_ rel *P*_*o*_) is the average of the differences between the logarithm of *P*_*o*_ following addition of β_1a_ subunit (log_10_ *P*_*oB*_) and the logarithm of the control *P*_*o*_ (log_10_ *P*_*oC*_), where *P*_*oC*_ was measured before β_1a_ subunit addition. **b** (*left*) Average relative mean open time (log_10_ rel *T*_*c*_). **c** (*left*) Average relative mean closed time (log_10_ rel *T*_*o*_) were calculated in the same way as the average log_10_ rel *P*_*o*_ (*above*). **a**–**c** (*right*) The average single-channel parameter values are shown *right* of the corresponding relative values. **a**–**c** Single-channel parameters were calculated from ~180 s of channel activity (at +40 and −40 mV). Data are shown for 0 nM β_1a_ (*black bar*), 10 nM β_1a_ subunit (*dark shade bar*), and 100 nM β_1a_ subunit (*light shade bar*), when examined. *Error bars* indicate ± SEM., *n* = 7−15 experiments/bar. *p < 0.05 vs control determined using paired (*left*) or un-paired (*right*) Student’s t-test, ^#^ *p* < 0.05 vs 10 nM β_1a_ subunit with RyR2 determined by ANOVA

As we reported previously [15], when added to the cytoplasmic *cis* chamber, the full-length β_1a_ subunit increases the activity of native RyR1 channels incorporated into planar lipid bilayers (Fig. 1a). Both 10- or 100-nM concentrations of β_1a_ subunit maximally activate RyR1 channels in the presence of 10 μM Ca^2+^ and 2 mM Na_2_ ATP [15]. The records in Fig. 1b show that RyR2 channel activity also increases upon cytoplasmic exposure to 10 nM β_1a_ subunit, but in contrast to RyR1, greater activation of RyR2 is observed with 100 nM β_1a_. On average, addition of 10 nM or 100 nM β_1a_ to the *cis* solution significantly increased the relative *P*_*o*_ of RyR2 by 1.8-fold and 2.6-fold, respectively (Fig. 2a, left). Data is presented as average relative *P*_*o*_ which is the average of the logarithm to the base 10 of *P*_*o*_ of each individual channel in the presence of β_1a_, relative to the logarithm of the *P*_*o*_ of its internal control activity measured before application of β_1a_. Use of relative *P*_*o*_ eliminates any effect of the normal variability between individual RyR channels [39, 42]. The logarithm is used to reveal the extent of variation of the effects of β_1a_. The average of the *P*_*o*_ parameter values are also shown to indicate absolute level of each parameter (Fig. 2a–c, right), however, the relative changes should be used as the most accurate indicator of effects of β_1a_ on RyRs. The effects on RyR2 channel activity were similar at +40 and −40 mV (relative *P*_*o*_ with 10 nM β_1a_ increasing by ~2-fold at +40 mV and ~1.7-fold at −40 mV), and these values were combined in the average data in Fig. 2a. It has been established that the activation of RyR1 by β_1a_ is maximal at 10 nM and does not increase between 10 and 1000 nM [15]. Therefore, the reduced efficacy of 10 nM β_1a_ on RyR2 suggests that affinity of RyR2 for β_1a_ is lower than that of RyR1.

The action of β_1a_ on single-channel gating parameters (Fig. 2b, c) reflected the changes in *P*_*o*_ (Fig. 2a, left and right). Both RyR1 and RyR2 activity increased with 10 and 100 nM β_1a_ as a result of increases in mean channel open time and an abbreviation of mean channel closed time (Fig. 2b, c). There was also a trend towards a greater increase in mean open time in RyR2 with the higher β_1a_ concentration that is consistent with the greater RyR2 open probability in the presence of 100 nM β_1a_. In contrast, RyR1 mean open time was similar at both β_1a_ concentrations. Mean closed times were similarly reduced for both RyR isoforms by 10 and 100 nM β_1a_.

**Fig. 3:**
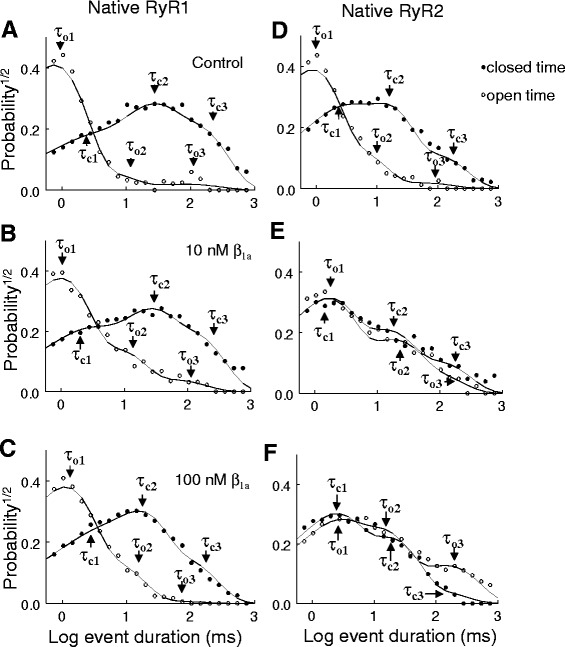
Effects of β_1a_ subunit on the distribution of representative RyR channel open and closed dwell times. Exponential open and closed time constants determined for RyR1 (**a**–**c**) and RyR2 (**d**–**f**). Open and closed times were collected into logged bins and the square root of the relative frequency of events (probability^1/2^) was plotted against the logarithm of open (*open circles*) or closed times (*filled circles*) in milliseconds. Examples are shown for the data from representative individual channels under control (**a**, **d**) and after exposure to 10 nM β_1a_ subunit (**b**, **e**) and then 100 nM β_1a_ subunit (**c**, **f**). The *solid lines* represent the fit of multiple exponentials to the data. The individual open time constants (*τ*_*o*1_, *τ*_*o*2_, and *τ*_*o*3_) and individual closed time constants (*τ*_*c*1_, *τ*_*c*2_, and *τ*_*c*3_) are indicated by *arrows*

**Fig. 4:**
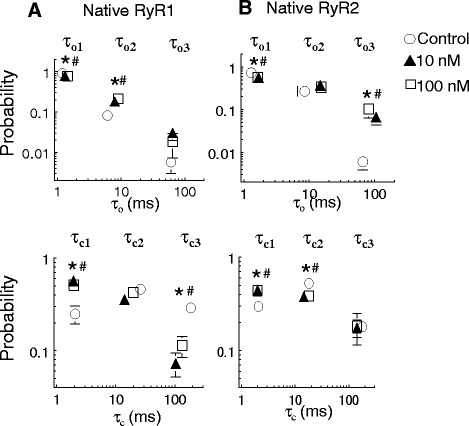
Effects of β_1a_ subunit on the distribution of RyR channel open and closed dwell times. The probability of open and closed events falling into each time constant is plotted against the respective time constant. The open (*τ*_*o*_, *top graphs*) and closed (*τ*_*c*_, *bottom graphs*) time constants and the probability of events in each time constant component were calculated from ~180 s of single channel activity (at +40 and −40 mV). Data is shown for **a** RyR1 and **b** RyR2 before (*open circle*) and after addition of 10 nM β_1a_ subunit (*open triangle*) or 100 nM β_1a_ subunit (*open square*), *n* = 6–12 channel traces. *Error bars* indicate ± SEM. The individual open time constants (*τ*_*o*1_, *τ*_*o*2_, and *τ*_*o*3_) and individual closed time constants (*τ*_*c*1_, *τ*_*c*2_, and *τ*_*c*3_) are indicated on the *top* and *bottom graphs*, respectively. *p < 0.05 vs the probability of events in each time constant in control with 10 nM β_1a_ subunit, determined by ANOVA. ^#^ *p* < 0.05 vs the probability of events in each time constant in control with 100 nM β_1a_ subunit, determined by ANOVA

The effects of β_1a_ on the open (*τ*_*o*_) and closed (*τ*_*c*_) time constant components and the relative distribution of events between time constants is presented in Figs. 3 and 4. Open events in RyR1 and RyR2 channels were well described by the sum of three time constants of ~1 (*τ*_*o*1_), ~10 (*τ*_*o*2_), and ~100 ms (*τ*_*o*3_) (Fig. 3). Closed times were also characterized by three time constants of ~1 (*τ*_*c*1_), ~10 (*τ*_*c*2_), and ~100 ms (*τ*_*c*3_) (Fig. 3). Figure 4 shows plots of the average probability of open (Fig. 4a, b, upper plots) and closed (Fig. 4a, b, lower plots) events as a function of the average time constant in the absence (control) and presence of either 10 or 100 nM β_1a_. Neither the time constants nor the relative probability of events for each time constant varied significantly (*p* = 0.12–0.99) between +40 and −40 mV and thus were combined in the average data.

Both 10 and 100 nM concentrations of β_1a_ subunit decreased the fraction of RyR2 openings in *τ*_*o*1_ by 18.7 ± 1.8 % (*p* = 0.003) and 16.3 ± 2.0 % (*p* = 0.012), respectively (Fig. 4b). There was a corresponding increase in the fraction of events for the longer open time constant components at both β_1a_ concentrations (Fig. 4b). In contrast to RyR2, the maximal increases in RyR1 activity after exposure to 10 or 100 nM β_1a_ subunit were reflected in a reduction in the fraction of RyR1 open events in *τ*_*o*1_ and increases in events in the longer time constant group (*τ*_*o*2_) at both β_1a_ concentrations (Fig. 4a). The closed time constant distributions in RyR2 and RyR1 were also altered by both 10 and 100 nM β_1a_, albeit in slightly different ways. There was an apparent transfer of 14.9 ± 3.1 % of closed events in RyR2 from *τ*_*c*2_ to *τ*_*c*1_ with 10 nM β_1a_ and 13.8 ± 4.7 % with 100 nM β_1a_ (Fig. 4b). In contrast, for RyR1, there were fewer long closed events in *τ*_*c*3_ and more short closed events in *τ*_*c*1_ with both 10 and 100 nM β_1a_ than in control (Fig. 4a).

Overall, the results indicate that 10 and 100 nM β_1a_ increase both RyR1 and RyR2 activity but with a reduced activation of RyR2 by 10 nM β_1a_. The dwell-time distributions indicate subtle differences between RyR1 and RyR2 in the effects of β_1a_ in redistribution between the different time constant components. In particular, β_1a_ induced a significant increase in events in the longest open time constant component in RyR2 but not RyR1 activity, while significantly reducing the number of events in the longest closed time constant component of RyR1 but not RyR2 activity.

#### The alternatively spliced ASI residues impact the functional interaction between β_1a_ and RyR1

**Fig. 5:**
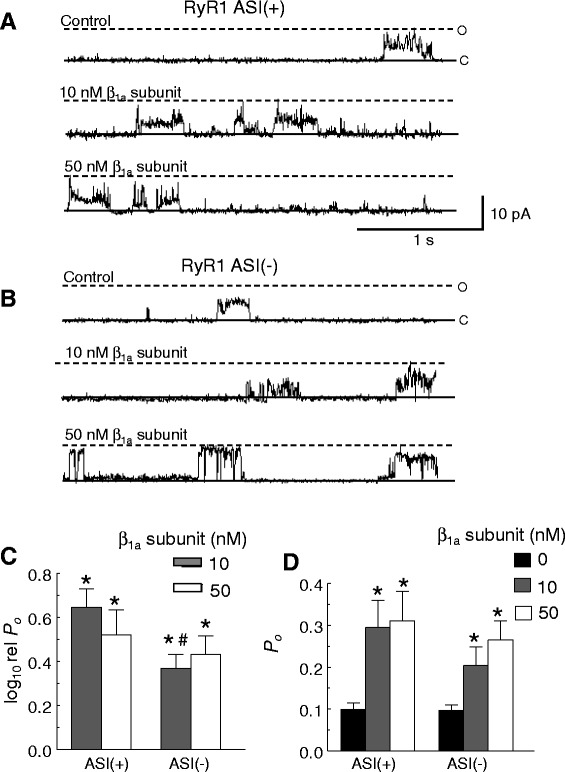
ASI residues enhance the effect of β_1a_ on recombinant RyR1 channel activity in lipid bilayers. **a**, **b** Three second (3 s) traces of ASI(+)RyR1 (**a**) or ASI(−)RyR (**b**) activity at +40 mV, opening upwards from the closed (*c*) to maximum open (*o*) level, before (*top panel*; control, *cis* 10 μM [Ca^2+^], no ATP) and after addition of 10 nM β_1a_ subunit (*middle panel*) or 50 nM β_1a_ subunit (*bottom panel*) to the *cis* chamber. **c** Average relative *P*_*o*_ (log_10_ rel *P*_*o*_) were calculated in the same ways as described for averaged relative *P*_*o*_ in Fig. 2a, left. **d** Average *P*_*o*_. **c** and **d** Single channel parameters were calculated from ~180 s of channel activity (at +40 and −40 mV). Data in **d** is shown for 0 nM β_1a_ (*black bar*), 10 nM β_1a_ subunit (*dark grey bar*), and 50 nM β_1a_ subunit (*light grey bar*). Error bars indicate + SEM, *n* = 9–12 experiments/bar. *p < 0.05 vs control or 0 nM β_1a_ subunit determined using paired (**c**) or un-paired (**d**) Student’s *t*-test, ^#^ *p* < 0.05 vs 10 nM β_1a_ subunit on RyR1 ASI(+) determined by ANOVA

There is a curious similarity between the cardiac RyR2 isoform and the ASI(−) splice variant of RyR1 in that both lack ASI residues. This may be relevant to the effect of β_1a_ on RyR1 and its contribution to EC coupling as we have shown that the presence of the alternatively spliced AS1 residues influences the gain of EC coupling in skeletal myotubes [27] and modulates RyR1 activity in vitro [28]. Therefore, we determined the impact of the alternatively spliced ASI residues on the activation of RyR1 by β_1a_. Recombinant ASI(-)RyR1 and ASI(+)RyR1 constructs [28, 43] were incorporated into lipid bilayers and the actions of the β_1a_ subunit on channel activity examined (Fig. 5). The ASI(+) isoform is the adult isoform of RyR1 and its sequence is equivalent to the adult rabbit RyR1 used in the previous section and to the cloned wild type (WT) rabbit RyR1 sequence described in the following section. It is notable in the single-channel activity, as shown in Fig. 5a, b (and in Fig. 7 below), that the recombinant channels (both ASI(-)RyR1 and ASI(+)RyR1) display strong sub-conductance (or sub-state) activity, with long channel openings to levels at ~50 % of the maximal conductance. Channel activity was measured as usual (“Methods” section) with an open threshold set at ~20 % of the maximum single-channel conductance to exclude baseline noise but to include sub-conductance openings to levels >20 % of the maximum. It is important to note that similar amounts of sub-conductance activity were seen in HEK293-expressed WT and ASI(−) compared in Fig. 5 and in WT and RyR1 K-Q channels compared in Fig.  8.  Similarly, the smaller amounts of sub-conductance activity were comparable in RyR1 and RyR2 isolated from muscle tissue and compared in Fig. 1. In each case, sub-state activity was similar in constructs being compared.

Subconductance activity has been associated with full or partial depletion of FKBP12 from RyRs [29, 30]. Densitometry measurements of immunoprobed RyR and FKBP following co-immunoprecipitation of the RyR1 complex indicate a 65 % reduction (*p* = 0.014) in FKBP bound to RyR1 in recombinant ASI(+) RyR1 when compared to native RyR1 isolated from muscle. Thus, the sub-conductance activity observed for the recombinant channels is consistent with reduced FKBP12 expression in HEK293 cells and reduced amounts associated with the recombinant RyR1 channels.

Cytoplasmic addition of 10 nM β_1a_ to ASI(+)RyR1 channels produced a significant ~4.4-fold increase in relative *P*_*o*_ and a significantly smaller ~2.3-fold increase in relative *P*_*o*_ of ASI(-)RyR1 (Fig. 5c). There was no significant difference between the degree of activation of the two RyR1 splice variants following application of 50 nM β_1a_ (Fig. 5c), so that the efficacy of 10 nM β_1a_ on ASI(−)RyR1 isoform appears to be less than that on ASI(+)RyR1. Therefore, the responses of both ASI(-)RyR1 and RyR2 that lack the ASI sequence to application of 10 nM β_1a_ are significantly reduced compared with RyR proteins that contain the ASI sequence, i.e., ASI(+)RyR1 or adult RyR1 isolated from rabbit skeletal muscle.

### The impact of the polybasic K3495-R3502 residues on EC coupling and β_1a_ activation of RyR1

#### The RyR1 polybasic motif facilitates EC coupling in expressing dyspedic myotubes

The PMB (residues K3495-R3502) in RyR1, located immediately downstream from the ASI region (A3481-Q3485), has been implicated in β_1a_ binding to RyR1 and EC coupling [18]. To assess the effect of the PMB on the interaction between β_1a_ and RyR1 channels in bilayers, a mutant of RyR1 in which all six polybasic residues were substituted with glutamines (RyR1 K-Q) was constructed. The functional effects of the RyR1 K-Q mutant on voltage-gated SR Ca^2+^ release and DHPR L-type currents were confirmed following expression in *dyspedic* myotubes [18].

**Fig. 6:**
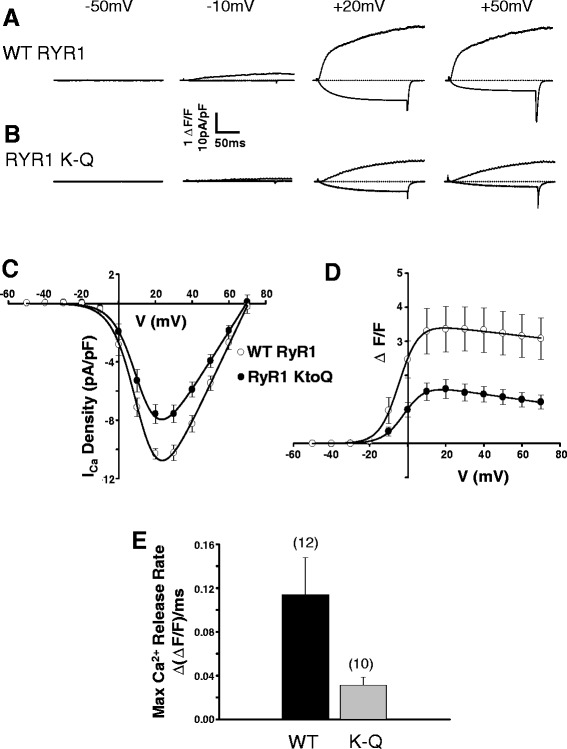
PBM mutation diminishes depolarization-induced SR Ca^2+^ release and DHPR Ca^2+^ currents in dyspedic myotubes. Dyspedic myotubes were transfected with either WT RyR1 or RyR1 K-Q mutant. **a**, **b** Representative L-type currents (*lower trace*) and Ca^2+^ transient (*upper trace*) obtained following depolarization to the indicated potentials of dyspedic myotubes expressing either **a** WT RyR1 or **b** RyR1 K-Q mutant. **c** Voltage dependence of average (±SEM) peak L-type Ca^2+^ current density (pA/pF) as a function of voltage. The data were fit (*continuous line*) with a modified Boltzmann function. **d** Voltage dependence of average (±SEM) peak Ca^2+^ transient amplitude as a function of voltage. The data were fit (*continuous line*) with a Boltzmann function. **c**, **d** Average (±SEM) values of the parameters from individual fits to each myotube are shown in Table 1. **e** The average (±SEM) peak of the first derivative of the fluo-4-fluorescence trace elicited during the test depolarization at 30 mV. **c**–**e** *n* = 10–12 myotubes

Depolarization-dependent Ca^2+^ release was measured simultaneously with DHPR L-type Ca^2+^ currents (Fig. 6a, b). Peak L-current density (Fig. 6c) and maximal DHPR Ca^2+^ conductance (*G*_max_) were significantly reduced in RyR1 K-Q mutant-expressing myotubes compared to WT RyR1-expressing myotubes (Table 1). Consistent with an earlier report [18], maximal voltage-induced SR Ca^2+^ release was also significantly reduced in RyR1 K-Q mutant-expressing myotubes (Fig. 6d and Table 1). In addition, the maximum rate of depolarization-induced Ca^2+^ release (approximated from the peak of the first derivative of the fluo-4 fluorescence trace elicited during a test depolarization at 30 mV) was significantly reduced in RyR1 K-Q-expressing myotubes compared to WT RyR1-expressing myotubes (Fig. 6e). These findings indicate that the RyR1 K-Q mutation substantially reduces voltage-induced SR Ca^2+^ release, with a small effect on maximal L-channel conductance. It should be noted that the reduced Ca^2+^ release is unlikely to result from reduced expression of RyR1 K-Q as it was previously shown that peak 4-chloro-m-cresol stimulated SR Ca^2+^ release was similar in WT RyR1- and RyR1 K-Q-expressing myotubes [18]. In addition, we found that WT RyR1 and RyR1 K-Q exhibited a similar punctate pattern and DHPR co-localization in expressing myotubes, consistent with similar levels of WT and K-Q expression and junctional localization (Additional file 1: Figure S1 and Additional file 2).

The small reduction in *G*_max_ is unlikely to fully account for the large reduction in voltage-induced SR Ca^2+^ release observed in RyR1 K-Q-expressing myotubes (Fig. 6d). This is supported by the sigmoidal voltage dependence of peak Ca^2+^ release, a feature of skeletal-type EC coupling demonstrating that Ca^2+^ release is independent of Ca^2+^ influx. The reduction in depolarization-induced DHPR currents and SR Ca^2+^ release could have resulted from poor targeting of the RyR1 K-Q mutant to the triad junction. However, double immunofluorescence labeling of RyR1 and the DHPR α_1_ subunit in expressing myotubes indicates that the DHPR and RyR1 proteins similarly co-localized as indicated by the yellow puncta in the overlays shown in Additional file 1. Therefore, compared to WT RyR1, the efficacy of voltage-induced SR Ca^2+^ release is reduced in RyR1 K-Q-expressing myotubes.

#### The polybasic motif in RyR1 is required for β_1a_ activation of RyR1

We explored the possibility that the reduction in efficiency of depolarization-induced Ca^2+^ release was due to an effect of the K-Q substitution on gating properties of the RyR channel or to its response to cytoplasmic [Ca^2+^] or ATP. RyR1 K-Q mutant channels exhibited unitary conductance of 222.3 ± 18.5 pS at +40 and 247.3 ± 33.1 pS at −40 mV, similar (*p* = 0.07–0.78) to that of WT RyR1 (311.9 ± 25.2 pS at +40 and 236.5 ± 12.5 pS at −40 mV), or ~220 pS as previously reported for WT RyR1 expressed in HEK293 cells under the recording conditions used in this study [44].

**Fig. 7:**
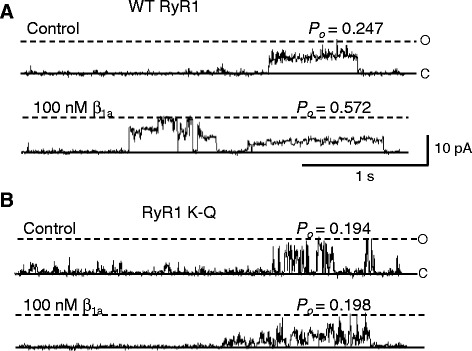
The K-Q mutation abolishes β_1a_ activation of RyR1 activity. **a**, **b** Three second (3 s) traces of WT RyR1 (**a**) or RyR K-Q mutant (**b**) activity at +40 mV, opening upward from the closed (*c*) state to the maximum open (*o*) level, before (*top panel*; control, *cis* 10 μM [Ca^2+^] and 2 mM ATP) and after addition of 100 nM β_1a_ subunit (*bottom panel*) to the *cis* chamber. Open probability (*P*_*o*_) is shown at the *right hand corner* of each trace

The effects of cytoplasmic Ca^2+^ and ATP were similar (*p* = 0.356–0.894) between +40 and -40 mV, and the data were combined. A decrease in *cis* free [Ca^2+^] from 1 mM to 10 μM caused a 1.7-fold increase in WT RyR1 *P*_*o*_ and a similar 1.6-fold increase in RyR1 K-Q *P*_*o*_, (log_10_ rel *P*_*o*_ of 0.22 ± 0.06 [*p* = 0.013] and 0.20 ± 0.09 [*p* = 0.048], respectively, *n* = 7 for each). Similar increases in *P*_*o*_ with a decrease in *cis* free [Ca^2+^] from 1 mM to 10 μM have been reported previously for recombinant WT RyR1 channels in lipid bilayers [44] and for [^3^H]ryanodine binding to RyR1 [33]. Addition of 2 mM Na_2_ ATP to the *cis* solution increased WT RyR1 activity by 2.2-fold and RyR1 K-Q activity by 2.5-fold (log_10_ rel *P*_*o*_ of 0.34 ± 0.12 [*p* = 0.032] and 0.40 ± 0.14 [*p* = 0.037], respectively, *n* = 7 for each). As observed for recombinant WT RyR1, prominent sub-state activity was also observed for recombinant RyR1 K-Q channels (Fig. 7a, b). The similar conductance, sub-state activity, and regulation by Ca^2+^ and ATP between WT RyR1 and RyR1 K-Q channels indicate that the K-Q mutation does not markedly alter RyR1 function in the absence of the β_1a_ subunit.

**Fig. 8:**
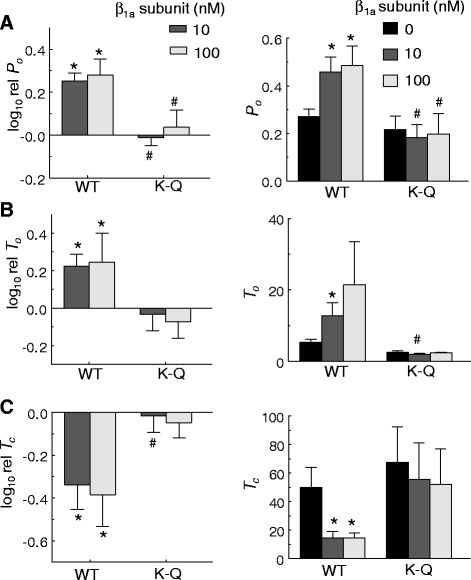
Effect of β_1a_ subunit on RyR1 in lipid bilayers is abolished for the K-Q mutation. **a–c** (*left*) Average relative *P*_*o*_ (log_10_ rel *P*_*o*_; **a**), mean open time (log_10_ T_o_; **b**) or mean closed time (log_10_ rel *T*_*c*_; **c**) were calculated in the same ways as described for averaged relative *P*_*o*_ in Fig. 2a, left. **a–c** (*right*) The average of the single channel parameter values shown to the right of the corresponding relative values. **a–c** Single-channel parameters were calculated from ~180 s of channel activity (at +40 and −40 mV). Data are shown without β_1a_ (0 nM β_1a_) (*black bar*), 10 nM β_1a_ subunit (*dark grey bar*), and 100 nM β_1a_ subunit (*light grey bar*), where applicable. *Error bars* indicate ± −SEM. *n* = 5–14 experiments/bar. *p < 0.05 vs control determined by paired (*left*) or un-paired (*right*) Student’s *t*-test, ^#^ *p* < 0.05 vs WT RyR2 determined by ANOVA

As before (Figs. 1, 2, 3, 4 and 5), *cis* addition of 100 nM β_1a_ significantly increased WT RyR1 channel activity (Fig. 7a). In marked contrast, the activity of RyR1 K-Q channels was unaffected by addition of 100 nM β_1a_ (Fig. 7a). As effects of the β_1a_ subunit on WT or RyR1 K-Q channels did not differ (*p* = 0.677–0.991) between +40 and −40 mV, values at these two potentials were again combined in the average data. On average, addition of 10 or 100 nM β_1a_ significantly increased WT RyR1 relative *P*_*o*_ by 1.8- and 1.9-fold, respectively (Fig. 8a), due to a significant increase in mean open time (Fig. 8b) and decrease in mean closed time (Fig. 8c). On the other hand, neither the relative *P*_*o*_ nor the mean open or closed times of RyR1 K-Q channels were significantly altered by addition of either 10 or 100 nM β_1a_ subunit (Fig. 8a–c). Thus, the ability of the β_1a_ subunit to activate RyR1 was abolished by neutralizing the polybasic residues within the K4395-R3502 region, indicating that the PBM is required for the functional effect of β_1a_ subunit on RyR1 activity.

## Discussion

The results presented here provide novel insight into the regions of RyR1 that influence the action of the β_1a_ subunit on RyR1 activity and have implications for the role of the β_1a_ subunit in skeletal muscle EC coupling. Our results demonstrate that the functional effect of 100 nM β_1a_ subunit is conserved between RyR1 and RyR2, although the activation by 10 nM β_1a_ was lower in RyR2 than in RyR1. Interestingly, a difference was also observed for the activation of ASI(−)RyR1 and ASI(+)RyR1 isoforms by 10 nM β_1a_, in that the lower concentration of β_1a_ was also less effective in activating ASI(−)RyR1 than ASI(+)RyR1. In contrast to the maintained, although different, activation of the two RyR isoforms by the β_1a_ subunit, neutralization of the PBM in RyR1 abolished β_1a_ activation of RyR1. One interpretation of this finding is that the ~50 % reduction in depolarization-dependent Ca^2+^ release results from disruption of direct β_1a_ activation of RyR1 during EC coupling.

### The action of β_1a_ subunit on RyR1 and RyR2 channel activity is largely conserved

The activation of RyR1 and RyR2 by β_1a_ suggests that the β_1a_ binding site is conserved across these RyR isoforms. The small concentration-dependent differences between effects on RyR1 and RyR2 suggest minor differences in either the binding residues or the binding pocket that reduces the affinity of β_1a_ for RyR2 (and ASI(−)RyR1). It is difficult to identify specific sequences that could account for the different affinities for β_1a_ as there is a 13.2 % (>600 residues) sequence disparity between RyR1 and RyR2 isoforms, according to a CLUSTALW multiple alignment [45] of rabbit RyR1 [Swiss-Prot: P11716.1] and rabbit RyR2 [Swiss-Prot: P30957.3]. Given that the string of positive residues is reduced from six to five, this variation is unlikely to account for the observed concentration-dependent difference between β_1a_ modulation of the two isoforms, although such a possibility cannot be fully excluded. Interestingly, except for one additional positive charge in the RyR1, the PBM is conserved in RyR2 (RyR1: K3495KKRR_ _ R3502 and RyR2: K3452*M*KRK_ _R3459) and thus is unlikely to account for the observed concentration-dependent difference between β_1a_ modulation of the two isoforms. However, just upstream from the PBM, four of the five ASI residues in RyR1 (A3481-Q3485) are missing from the rabbit (and predicted pig) RyR2 sequence. It may be significant that the lack of ASI residues in full-length ASI(−)RyR1 reduces the efficacy of 10 nM β_1a_-mediated activation. Thus, it is plausible that the difference between β_1a_ modulation of RyR1 and RyR2 is partially due to the presence or absence of the ASI residues, respectively. The conservation of the modulatory effect of β_1a_ on RyR1 and RyR2 does not reflect the in vivo studies showing that RyR2 is unable to replace RyR1 in skeletal muscle EC coupling [25, 46]. However, the lack of skeletal muscle EC coupling in RyR2-expressing dyspedic myotubes is most likely due to the fact that DHPR tetrads are not restored in RyR2-expressing dyspedic myotubes [46], indicating that β_1a_ is unable to correctly align DHPRs with RyR2 in order to ensure a direct interaction between the two proteins. It is also possible that the II-III loop critical region is unable to engage with RyR2 through β_1a_.

#### The importance of the RyR1 polybasic motif for β_1a_ subunit increase in RyR1 channel activity

The role of the RyR1 PBM in the β_1a_-mediated increase in channel activity was assessed from the response of recombinant RyR1 K-Q channels in bilayers to the addition of the β_1a_ subunit. RyR1 K-Q and WT RyR1 channel conductance and regulation by cytoplasmic modulators were similar, indicating that RyR1 K-Q channels function normally. However, RyR1 K-Q channel activity was unaltered by the β_1a_ subunit. Therefore, the reduction in voltage-gated Ca^2+^ release observed in RyR1 K-Q-expressing myotubes is likely to reflect a specific effect of the polybasic residues on β_1a_ subunit regulation of RyR1 channel activity during EC coupling rather than a general effect on RyR1 channel function. However, we cannot rule out the possibility that modest differences in RyR1 expression contribute to the reduced L-channel conductance and voltage-gated Ca^2+^ release in K-Q-expressing myotubes, although this seems unlikely given previous reports of 4-chloro-m-cresol stimulated SR Ca^2+^ release in myotubes RyR1 K-Q [18] and the data in Additional file 1.

Given that the PBM in the larger M3201-W3661 fragment of RyR1 is required for pull down of the β_1a_ subunit [18], it is likely that the lack of an effect of β_1a_ on RyR1 K-Q channels is due to the inability of β_1a_ to bind to the PBM mutant channel. Alternatively, the PBM may be important for maintaining RyR1 in a conformation permissive for β_1a_ binding, rather than directly contributing to binding, as the RyR1 basic residues would be unlikely to interact with the hydrophobic residues in the β_1a_ C-terminal domain (L496, L500, and W503) previously shown to bind RyR1 [21]. In addition, although the PBM is implicated in ASI-mediated inter-domain inhibition of RyR1 [27, 43], the structure of this motif is not altered by substituting three of the six basic residues with alanine residues [27]. Thus, neutralization of the PBM more likely disrupts the inter-domain interaction rather than changes the intrinsic structure of the ASI-polybasic region. In this case, disruption of the RyR1 PBM inter-domain interaction may alter an essential conformation of the β_1a_ binding site or prevent β_1a_ access to its binding site on RyR1.

The β_1a_ subunit is unlikely to be the sole signaling conduit between the DHPR and RyR1 during EC coupling. Consistent with this, expression of the RyR1 K-Q mutant in dyspedic myotubes partially restored sigmoidal, depolarization-dependent Ca^2+^ release even though β_1a_ modulation of RyR1 in bilayers was abolished. In addition, previous studies have also shown that truncation of β_1a_ C-terminal residues, essential for β_1a_ modulation of RyR1, also reduces but does not abolish depolarization-induced Ca^2+^ release [17, 40], an outcome that was also observed in adult skeletal muscle fibers that overexpressed a β_1a_ subunit interacting protein, Rem [20]. Finally, alanine substitution of β_1a_ subunit hydrophobic triplet residues (L496, L500, and W503) only partially reduces depolarization-induced Ca^2+^ release in β_1a_ null myotubes [14], despite this mutation fully abolishing β_1a_ modulation of RyR1 activity in vitro [21].

#### The role of the RyR1 ASI residues in β_1a_ subunit increase in RyR1 channel activity

It is curious that 10 nM β_1a_ subunit increased ASI(−)RyR1 activity less than ASI(+)RyR1 given that EC coupling is enhanced in dyspedic myotubes that express ASI(−)RyR1 relative to ASI(+)RyR1 [27]. The greater activation of ASI(+)RyR1 by 10 nM β_1a_ is consistent with effects reported previously of agonists of RyR1, including caffeine and 4-chloro-m-cresol [27, 28]. Thus, the increased gain of EC coupling observed for ASI(-)RyR1 may not reflect a contribution of the β_1a_ subunit to EC coupling. However, it is possible that activation of RyR1 by agonist binding includes a common mechanism for activation by agonists that differs from that involved in EC coupling. As it is likely that more than one interaction between RyR1 and the DHPR is involved in EC coupling, the combined result of these interactions may produce different effects on the two alternatively spliced variants such that ASI(−)RyR1 channels are activated more strongly by depolarization than ASI(+)RyR1 channels.

The possibility that the ASI region is involved in an inhibitory inter-domain interaction was previously investigated using peptides corresponding to the ASI region from T3471–G3500 [43]. The peptide corresponding to the ASI(−) sequence was more effective in activating ASI(-)RyR1 than ASI(+)RyR1. Together with the finding that ASI(−)RyR1 channels were generally less active than ASI(+)RyR1 channels, these findings suggest that stronger inhibitory inter-domain interactions may exist in ASI(−)RyR1. It is possible then that the triggering mechanism activated during EC coupling disrupts this inhibitory inter-domain interaction giving rise to greater activation of ASI(-)RyR1. This disruption may not occur with RyR1 agonist binding and indeed a stronger inhibitory inter-domain interaction in ASI(-)RyR1 may even oppose activation by β_1a_ and other agonists, allowing for these triggers to more strongly activate ASI(+)RyR1 channels.

## Conclusions

The results presented in this study suggest that a functional β_1a_ interaction is conserved between RyR1 and RyR2 and that β_1a_ activation of RyRs is regulated by the presence of the ASI residues. Importantly, we also show that the PBM residues are essential for direct activation of RyR1 by β_1a_ subunit in vitro. This suggests that the ~50 % reduction in Ca^2+^ release during EC coupling in dyspedic myotubes expressing RyR1 with a neutralized PBM is due to removal of β_1a_ activation of RyR1, and hence, that other DHPR-RyR1 coupling elements (e.g., II-III loop critical domain) contribute to transmission of the remaining Ca^2+^ release during EC coupling.

## Additional files

Additional file 1: Figure S1. Description of data: a figure with two parts showing immune-fluorescent labeling of DHPR and RyR1 in myotubes. Additional file 2:
**A legend to additional Figure S1.**

## Abbreviations

ASI: alternatively splicing region I
DHPR: dihydropyridine receptor
EC: excitation-contraction
PBM: polybasic motif
RyR1: skeletal ryanodine receptor
RyR2: cardiac ryanodine receptor
SR: sarcoplasmic reticulum
