# Supplementary figures and images for: Regions of ryanodine receptors that influence activation by the dihydropyridine receptor β1a subunit
Source: Skelet Muscle. 2015 Jul 22;5:23. doi: 10.1186/s13395-015-0049-3 (PMC4510890; doi:10.1186/s13395-015-0049-3)

## Slide 1
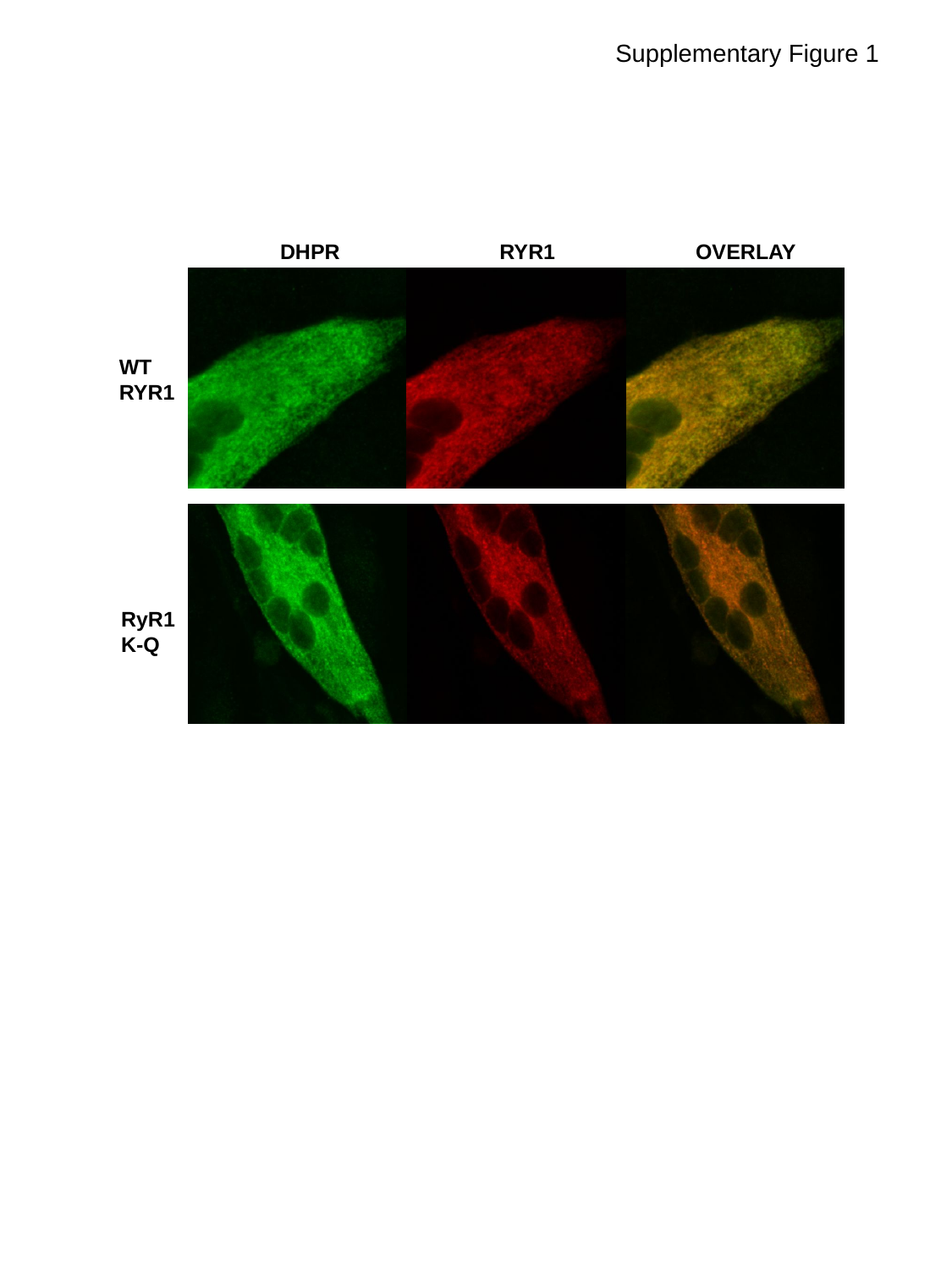

Supplementary Figure 1
DHPR
RYR1
OVERLAY
WT RYR1
RyR1 K-Q

Supplement: Additional file 1: Figure S1. — Description of data: a figure with two parts showing immune-fluorescent labeling of DHPR and RyR1 in myotubes. [file 13395_2015_49_MOESM1_ESM.pptx]
