# Supplementary material for: Regions of ryanodine receptors that influence activation by the dihydropyridine receptor β1a subunit
Source: Skelet Muscle. 2015 Jul 22;5:23. doi: 10.1186/s13395-015-0049-3 (PMC4510890; doi:10.1186/s13395-015-0049-3)
Supplement: Additional file 2: — A legend to additional Figure S1. [file 13395_2015_49_MOESM2_ESM.docx]

**Legend to additional File 1.** Double immunofluorescence labeling of DHPR (first column) and RyR1 (second column) in representative dyspedic myotubes expressing either WT RyR1 (*top* row) or K-Q RyR1 mutant (*bottom* row). Merged images (third column) demonstrate co-localization of endogenous DHPRs with expressed RyR1s in puncta (yellow foci).
